# ANNOUNCEMENTS & RESOURCES

**Published:** 2017-08-07

## Courses

### MSc Public Health for Eye Care, London School of Hygiene & Tropical Medicine

Ten fully funded scholarships are available for Commonwealth country nationals. The course aims to provide eye health professionals with the public health knowledge and skills required to reduce blindness and visual disability. For more information visit **www.lshtm.ac.uk/study/masters/mscphec.html** or email **romulo.fabunan@lshtm.ac.uk**

**Figure:**
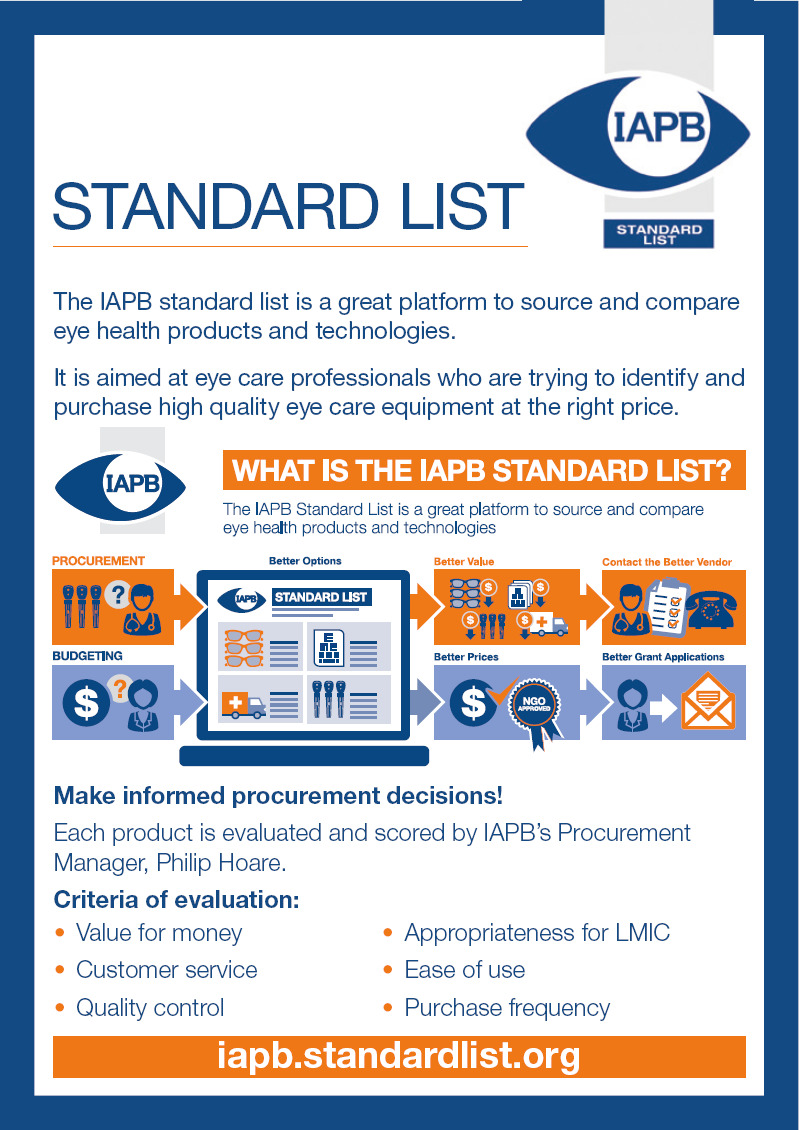


## Correspondence article: online only

**Figure:**
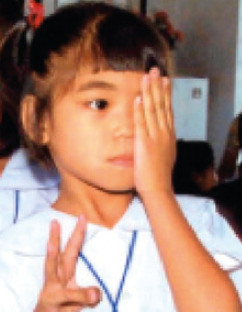


USAID Child Blindness Programme partners have identified areas that have the greatest impact on on project sucess, including recording gender data to ensure equitable access. Read more on **www.cehjournal.org/Lessons-from-the-USAID-Child-Blindness-Programme**

## Subscriptions

Contact Anita Shah **admin@cehjournal.org**

### Subscribe to our mailing list

**web@cehjournal.org** or visit **www.cehjournal.org/subscribe**

### Visit us online

**www.cehjournal.org www.facebook.com/CEHJournal https://twitter.com/CEHJournal**

